# Misinformation, infighting, backlash, and an ‘endless’ recovery; policymakers recount challenges and mitigating measures after a vaccine scare in the Philippines

**DOI:** 10.1080/16549716.2022.2077536

**Published:** 2022-08-05

**Authors:** Mark Donald C. Reñosa, Jonas Wachinger, Kate Bärnighausen, Vivienne Endoma, Jhoys Landicho-Guevarra, Jeniffer Landicho, Thea Andrea Bravo, Mila Aligato, Shannon A. McMahon

**Affiliations:** a Heidelberg Institute of Global Health, Ruprecht-Karls Universität Heidelberg, Heidelberg, Germany; b Department of Epidemiology and Biostatistics and Department of Health, Research Institute for Tropical Medicine, Muntinlupa, Philippines; c School of Public Health, University of the Witwatersrand, Johannesburg, South Africa; d International Health Department, Johns Hopkins Bloomberg School of Public Health, Baltimore, MD, USA

**Keywords:** Vaccines, immunization, vaccine hesitancy, health policy, policymakers, health system, Philippines

## Abstract

**Background:**

Vaccine scares undermine longstanding global health achievements. Remarkably little data has documented the lived experiences of policymakers working amidst vaccine scares and navigating their fallout. As a result, chances and challenges of large-scale national recuperation efforts are poorly understood.

**Objective:**

This study aims to explore the perspectives of policymakers involved in ongoing efforts to boost vaccine confidence in the Philippines following a 2017 Dengvaxia scare and the current COVID-19 pandemic.

**Methods:**

Between August and November 2020, we conducted 19 semi-structured narrative interviews with purposively selected policymakers from governmental agencies and non-governmental organizations in the Philippines. Interviews were conducted online, transcribed, and analyzed following the tenets of reflexive thematic analysis.

**Results:**

We present results as an emerging model that draws on a chronology conveyed by policymakers in their own words. The Dengvaxia scare proved ‘a decisive wedge’ that splintered Filipino society and pitted governmental agencies against one another. The scare stoked distorted vaccination narratives, which were ‘accelerated rapidly’ via social media, and ignited feelings of uncertainty among policymakers of how to convey clear, accurate health messaging and how to prevent drops in care-seeking more broadly.

**Conclusions:**

Efforts to regain trust placed exceptional burdens on an already-strained health system. Respondent-driven recommendations on how to reinforce vaccine confidence and improve vaccination rollout include: developing clear vaccine messages, fostering healthcare providers’ and policymakers’ communication skills, and rebuilding trust within, toward and across governmental agencies. Further research on how to build enabling environments and rebuild trust in and across institutions remains paramount.

## Background

Vaccine scares, defined as highly publicized discourses on vaccination safety and efficacy, have eroded trust in vaccines in several countries [1,2]. Current literature highlights that public exposure to vaccine scares can affect public opinion not only regarding the specific vaccine in question, but also regarding vaccination in general, resulting in long-term public health consequences [3–5]. Examples of recent vaccine scares and their fallout include declines in measles, mumps, rubella (MMR) vaccine uptake among children in the UK following a retracted article about the risk of autism after vaccination [6], and drops in general public confidence on human papillomavirus (HPV) vaccine following reports of serious adverse effects in Japan and Denmark [7,8]. Studies in China and Australia have similarly highlighted how news of a sudden vaccine recall can foster vaccine hesitancy (VH; defined as the delay or refusal despite their availability) in the general public [9,10].

Following vaccine scares, personnel engaged in vaccine promotion often find themselves in the challenging position of having to negotiate the scare’s fallout – including a proliferation of misinformation – while continuing to address pre-existing, broader challenges in vaccination rollout [11–13]. Several studies have outlined experiences of frontline medical personnel, such as doctors and community health workers, who encounter this fallout in their daily encounters with clients [14–16]. However, there is limited research examining how policymakers perceive, react and – in the longer term – respond to fallout-associated challenges.

Literature on policymakers’ perspectives amid other acute, health-related scares (beyond vaccination) has highlighted that resulting policies are not only informed by scientific evidence but also influenced by sociocultural dynamics and community norms [17–19]. In a qualitative study in Kenya, researchers underscored the challenges in policy development and implementation in general, particularly in the formulation of tobacco control policies [18]. Similarly, a study among Filipino policymakers on the topic of national-level governance found that several factors inhibited policy development including fragmented leadership and limited multi-sectoral collaboration, which slowed the implementation of a reproductive health law [19]. While such literature highlights how policymakers perceive challenges and facilitators amid an overarching policymaking process, information that highlights lived experiences amid a broader health-related scare, its fallout, and recuperation is lacking. This dearth of evidence is particularly prominent in the context of vaccine scares.

Policymakers involved in formulating health-related policies often encounter public and institutional pressures (including, but not limited to, perceived urgency of the issue) [17,20,21]. Scholars have also underscored that policymaking involves policymakers incorporating their own values and beliefs driven by their internalized and externalized perspectives such as their interests (how they think the world should work), ideology (how they would like the world to work) and beliefs (based on their knowledge, how the world actually works) [17,21]. The processes underlying policymaking can be structured in three main phases: Understanding the challenge that impacts the public (agenda setting), developing policy options (policy formulation and decision making), and reflecting the sentiments and values of the affected parties (policy implementation and evaluation) [20].

While existing work has focused on how vaccine scares are experienced by the general public [22], qualitative exploration of how these scares have shaped narratives about vaccines, health programming, and the health system in general in the eyes of policymakers in the Philippines is lacking.

In this study, we explore the perspectives of policymakers in the Philippines who are involved in ongoing public health efforts to rebuild and expand vaccination efforts after a vaccine scare: In 2017, new evidence suggested that the Dengvaxia vaccine, a novel dengue vaccine that had been rolled out on a large scale in the country for over a year, posed previously unknown side effects. This announcement resulted in a vast and highly politicized controversy, followed by plummeting vaccination rates [23]. We also present policymaker perspectives regarding how the current COVID-19 pandemic poses challenges and opportunities to health education and vaccination efforts in a context where public trust in vaccines has been recently challenged. With our findings, we aim to present respondent-driven guidance on how to rebuild vaccine confidence, reinforce vaccine program resilience, and improve future vaccination rollout in the Philippines and similar contexts.

## Methods

### Study design

This qualitative study is part of a larger mixed-methods study designed to develop and test a story-based VH intervention to revive vaccine confidence in the Philippines. Detailed information regarding overall study design and procedures are published elsewhere [24].

### Study setting

The Republic of the Philippines is an archipelago in Southeast Asia, spread across more than 7,000 islands and home to more than 109 million people [25]. The country is one of several low- and middle-income countries (LMICs) that recently experienced an erosion of public trust in childhood vaccinations [26]. With the country’s geographic makeup and decentralized health system, programmatic issues relating to logistics, workforce, and service delivery (inclusive of vaccines) remain a challenge [27]. Moreover, an inadequate workforce to cover and monitor large or densely populated areas, along with health-care workers’ (HCWs) overwhelming responsibilities have undermined past efforts to address VH [27].

In April 2016, the Philippines Department of Health (DOH) launched Dengvaxia (a dengue vaccine developed and produced by Sanofi Pasteur) as part of the school-based immunization program for children aged 9–14 years in those regions of the country most severely affected by dengue [23]. In November 2017, after more than a year of vaccine rollout, Sanofi Pasteur released an interim analysis indicating that the vaccine could increase the risk of developing more severe forms of dengue in children who had not been previously infected with the virus, and the company asked regulators to update their product label [23]. In the Philippines, a press release outlining the possible additional risk sparked panic among the general public [23,28]. The new Philippines government, which in 2016 had succeeded the previous government that had first implemented Dengvaxia, first suspended the rollout of Dengvaxia in December 2017 and later, in February 2019, banned the vaccine nationally.

In a viral Facebook post, a blogger described Dengvaxia as a ‘genocide against Filipino children’, a phrase that gathered traction in traditional and social media and further fueled public uproar [29]. Shortly after the misinformation circulated on Facebook, allegations surfaced that Dengvaxia was linked to the death of a Filipino child, which the Philippines Public Attorney’s office investigated via a televised autopsy; other sources argued that the child had a pre-existing condition [22,23,29]. Despite many mitigating measures such as nationally televised dialogues, the creation of hotlines and close monitoring of Dengvaxia recipients, a widespread vaccine scare engulfed the country. Several vaccine experts have affirmed the scientific soundness of the interim report stating that Dengvaxia poses an increased risk for seronegative individuals [30]. However, claims by some government officials that vaccinated children died due to a corrupt previous government’s adoption of Dengvaxia represented an example of a ‘weaponization’ of Dengvaxia [22,23,29,31]. Ultimately, vaccine confidence and vaccination rates plummeted in the years following the scare [2], leading to measles and polio outbreaks in 2019 [32,33].

### Data collection

Between August and November 2020, we conducted 19 semi-structured narrative interviews with purposively selected policymakers who are formally involved in the field of vaccination. We define policymakers as those who are involved in making policies and policy decisions. We initially invited respondents via email or phone call and performed a one-on-one phone call where the study was briefly introduced. After the initial call, we arranged an appointment to further explain the study, answer any questions, request consent, and proceed for a formal interview [24,34]. Policymakers included National Immunization Program managers, coordinators, team leaders from government agencies (DOH, Department of Education (DepEd)) and non-government organizations (World Health Organization (WHO) country and Western Pacific region offices, and UNICEF). We excluded policymakers who had less than a year of experience in their role and those who were not actively engaged in the field of vaccination. Two policymakers we had originally approached declined to participate citing competing priorities and busy schedules due to the COVID-19 pandemic.

**Figure 1.**
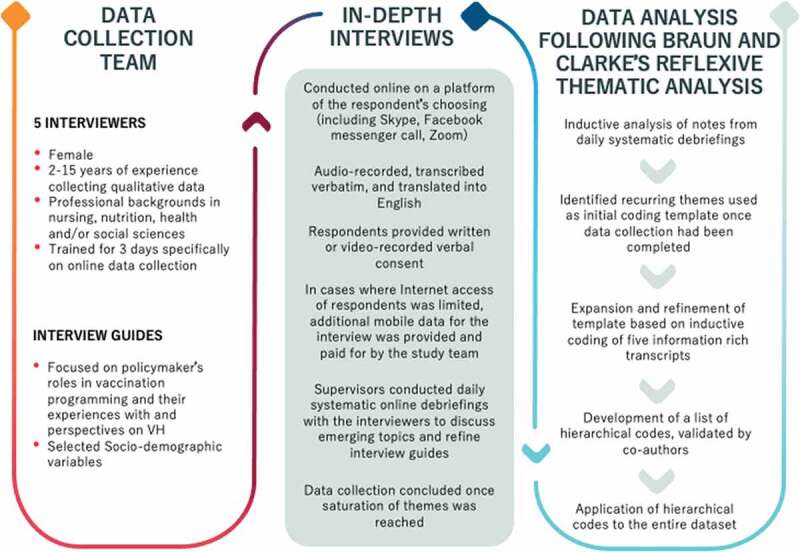
Description of study recruitment, data collection and analysis.

Five trained data collectors conducted narrative interviews online with the 19 policymakers agreeing to participate after obtaining informed written or video-recorded verbal consent (see Figure 1 for detailed information on interviewer profile and data collection procedures). We conducted daily systematic online debriefings to discuss emerging topics and refine interview guides [35]. Data collection concluded once saturation was reached.

### Data analysis

All audio- or video-recorded interviews were transcribed verbatim and translated into English by bilingual research assistants, and transcribed in accordance with qualitative standards [36]. Our data analysis was guided by the tenets of reflexive thematic analysis as outlined by Braun and Clark (see Figure 1) [37]. During data collection, we started to inductively analyze notes from daily systematic debriefing sessions [35]. By the time data collection was complete, several recurring themes had been identified, and were used as a preliminary coding template. We further expanded and restructured this template following line-by-line analysis of transcripts. The lead author initially conducted inductive coding of five information-rich transcripts to develop a list of hierarchical codes which were then applied iteratively to the entire dataset using NVivo 12 Pro (QSR International Pty Ltd. Version 12, 2018). The lead author routinely provided analytical summaries to co-authors (primarily JW and KB) and received feedback for further refining and finalizing the work.

### Reflexivity

The Dengvaxia scare and its fallout are politically charged topics in the Philippines. The sensitivity of the political context also emerged in our interactions with respondents, who served on the forefronts of vaccine confidence recuperation efforts. To increase the likelihood of receiving diverse and forthright perspectives on the issue and to triangulate our data, we recruited respondents from a broad range of policymaking organizations, both directly affiliated with the Philippines government and from non-governmental sectors.

All interviewers (VE, JLG, JL, TAB, MA) and the lead author (MDCR) are Filipino nationals currently empoyed at the Research Institute of Tropical Medicine (RITM), which is the research arm of the Philippines DOH for public health research. Our work at Communications Officer RITM has exposed us to the deep-seated realities of policymaking and the intricacies of implementing health programs in urban and rural communities – factors that in some way influence how we view research, interventions, data, and findings. We note that RITM was involved in the Dengvaxia clinical trial in 2011, however none of the researchers for this study were involved in this trial or the Dengvaxia rollout (and have not been involved in any vaccine-related trials for the past 8 years); the research team did not feel beholden to the Dengvaxia studies. While RITM’s role may have resulted in a desirability bias (in some instances more complementary perspectives, in other instances more critical perspectives), we sought to mitigate this by emphasizing the privacy and confidentiality of the data prior to and during interviews, and we highlighted that an honest reflection on professional experiences could help to mitigate future challenges. Consistent assurances may have reduced biases and bolstered trustworthiness.

## Results

A majority of the 19 policymakers interviewed were female (n=14) and had professional backgrounds as medical doctors (n=16) with the rest trained as communications officers (n = 2) or nurses (n = 1; see Table 1). Respondents had 1–29 years (median: 5 years) of experience as a policymaker in the field of vaccination.

**Table 1.** Demographic profiles of the respondents.

Characteristics	(n = 19)	%
Sex		
Male	5	26.3%
Female	14	73.7%
Civil Status		
Single	7	36.8%
Married	12	63.2%
Age Group		
<30 years	1	5.3%
30–40 years	6	31.6%
41–50 years	5	26.3%
>51 years	7	36.8%
Cadre		
Medical Doctor	16	84.2%
Registered Nurse	1	5.3%
Communications officer	2	10.5%
Number of years working as policymaker (within a vaccination specific role)		
<10 years	13	68.4%
10–20 years	4	21.1%
>20 years	2	10.5%

While a majority of respondents were Filipino nationals, two respondents were foreigners with a longstanding presence as policymakers in the Philippines. We used the term ‘*Filipino policymakers*’ not as a description of respondents’ nationality, but to specify their role as policymakers living and working in the Filipino setting.

**Figure 2.**
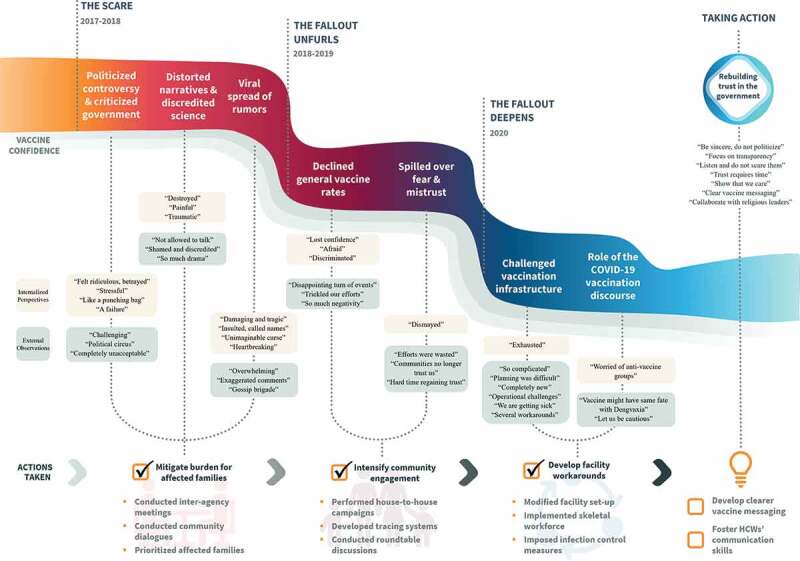
Policymaker’s perspectives on a vaccine scare and actions taken to bolster vaccine uptake in the Philippines.

Policymakers – regardless of their position, gender, or years of professional experience – consistently described an immutable process they experienced in the face of acute vaccine-related discourses: 1) *The scare* – the case of Dengvaxia; 2) *The fallout unfurls* – domino and spillover effects; 3) *The fallout deepens* amid the COVID-19 pandemic; 4) Taking action – *policymakers’ perspectives on rebuilding trust and vaccine confidence* (see Figure 2). The model also reflects three essential concepts that contribute to policymaking (particularly related to agenda setting): *external observation, internalized perspective* and *actions taken*. The external observation (how policymakers perceive the situation) and their internalized perspectives (how policymakers internalize what is happening) are presented as inextricably intertwined concepts, which directly informed actions taken. For each theme, we present key quotes with the respondent’s educational background (MD = Medical Doctor, RN = Registered Nurse, CO = Communication Officer) and their duration working as policymakers as identifiers.

### The scare – the case of Dengvaxia

#### Loss of trust, distorted narratives, and personal guilt

Respondents recounted challenges to vaccination promotion that they had faced in the past – from rumors related to infertility and a tetanus toxoid vaccine, to natural calamities (typhoons or volcanoes) that inhibit vaccine rollouts – but they described the Dengvaxia scare as a ‘*reeling event*’ [MD, 16 years] that overwhelmed the entire vaccination program. Respondents recalled how tragic they had found the Dengvaxia scare, particularly the manner in which it was swiftly politicized. Respondents spoke at length of their pains and frustrations regarding the situation becoming a ‘*political circus*’ [MD, 16 years]. They felt that instead of presenting a united front to address public concerns, various governmental and non-governmental institutions attempted to instrumentalize the scare to suit differing political agendas.

Among the most painful fallouts of the Dengvaxia scare for DepEd policymakers was a loss of trust parents expressed in their children’s teachers and in the DepEd in general, who they saw as being responsible for putting their children at risk: ‘*Parents are very scared, and we are the ones they see. Fathers, even grandmothers, are angry at us*’ [MD, 16 years]. DepEd respondents also critiqued the DOH who in their eyes designed a faulty school-based vaccination program (sometimes against the explicit advice of DepEd representatives), and for letting the traditional media shift the blame to DepEd amid the scandal.

As the vaccine scare unfolded, respondents lamented how the senate (the upper legislative branch of the government) allowed those who lack training in medicine or public health to present distorted narratives and to discredit scientists, with one respondent describing how the Public Attorney’s office ‘*destroyed the integrity of the DOH and the medical profession*’ [MD, 16 years], especially maligning those DOH officials who favored and bolstered vaccine rollout. Respondents said that the general public was eager to hear a medical person speak the ‘*truth*’ [MD, 20 years], but that DOH medical staff involved in the Dengvaxia rollout often were not allowed to talk due to ongoing legal investigations, or that they were lacking the necessary information to respond to issues raised regarding Dengvaxia. One respondent who described Dengvaxia as ‘*a good vaccine*’ [MD, 16 years] vented that the DOH did not do its part to save the continuous roll-out of the vaccine but facilitated its demise.

In addition to these perceived shortcomings of government communication efforts, respondents also critiqued how media outlets allowed false narratives to proliferate: ‘*people mix up emotions over science … the media’s responsibility is to be the balance … to focus on truth, not the other way around*’ [MD, 20 years]. Instead, respondents recalled TV newscasts that showcased mothers holding pictures of their children and of other peoples’ children who allegedly died after Dengvaxia vaccination. On social media platforms, especially Facebook, respondents with a scientific background reported facing abuse and having their scientific rigor and reputation questioned. Respondents described feeling ‘*insulted, damaged and heartbroken*’ [MD, 2 years] seeing a viral spread of memes and misinformation about vaccines. Taken as a whole, policymakers described finding themselves being put ‘*in a bad light*’ [MD, 3 years] by the sentiment in lay and social media, and felt like they were ‘*gasping for air*’ [MD, 16 years] and ‘*running from a ticking bomb*’ as they became ‘*collateral damage*’ [MD, 16 years] in a broader political dispute among elected parties. When probed on how they navigated these tensions, respondents narrated moments of introspection where they sought to recall what initially drew them to public service, others used the experience as a reminder that there are always lessons to learn. Still others emphasized a need to take deliberate steps into the future rather than remaining focused on the past. Exemplary quotes include a desire to remember one’s ‘*passion for children’s welfare*’ [MD, 6 years], and to ‘*acknowledge the problem and that there is something to be done*’ [MD, 2 years].

Respondents also recalled how they personally were struggling with their own role during the vaccine scare. Some respondents felt that they had failed to safeguard the welfare of Filipino children, which they saw as their key responsibility. One DepEd respondent recalled an incident where a Dengvaxia-vaccinated child asked her: ‘*Doctor, am I going to die as well?*’ [MD, 6 years], which the respondent described as tragic and difficult to deal with, particularly in light of the absence of psychological support from the DOH during or after the controversy.

#### Actions taken: mitigate burden for affected families

During the height of the vaccine scare, respondents recalled several inter-agency consultative meetings (DOH, DepEd and other stakeholders including local government units), community dialogues with parents and affected families, and local health education campaigns. As a result of community dialogues, respondents described how the DOH proactively implemented extra services to support the health of children who had been vaccinated with Dengvaxia. This included priority treatment in all government hospitals via a ‘*dengue fast lane*’, additional health monitoring by city and municipal HCWs, and free access to vitamins and medicines.

However, respondents lamented that the additional benefits allotted to children who had been vaccinated were sapping resources originally intended for other child health programs. Respondents also felt that HCWs were ‘*bearing all the weight*’ [MD, 6 years] of the situation, and that retrospectively the DOH could have prevented some of the public uproar if they had emphasized ‘*right and clear vaccine messaging*’ [CO, 5 years] immediately after Sanofi Pasteur published the interim results on Dengvaxia risks.

### The fallout unfurls – domino and spillover effects

#### Spillover effects to other vaccine programs and beyond vaccination

Respondents from the DOH and the WHO (those stationed in the country and western pacific region offices) sensed that Dengvaxia scares increased VH, but that the hesitancy did not extend across *all* vaccines, but rather affected vaccines administered via community mobilization or school-based vaccination. Respondents described how parents would mention Dengvaxia when they were ‘*selectively rejecting*’ [MD, 20 years] vaccines (particularly the MMR vaccine). Respondents said they experienced difficulties rolling out the community-based MMR vaccination campaign soon after the vaccine scare, as mothers described fears that the campaign may be a ruse to again administer Dengvaxia. Respondents said that the Dengvaxia issue was still ‘*fresh in parents’ memories*’ [MD, 2 years], and continued to be a key driver of parental refusal and delays.

One key experience of the fallout for respondents was the Philippines losing its 20-year polio-free status in 2019 due to the rapidly declining vaccination rates, which respondents felt sad and ‘*deeply frustrated*’ [MD, 16 years] about. As one respondent described the years following the vaccine scare: ‘*The immunization coverage plummeted, which resulted in several outbreaks … now, our major challenge was really to address those outbreaks … like recovery is endless … and forever*’ [MD, 4 years]. Adding to the frustration, respondents lamented that parents are now rejecting health interventions implemented at schools that did not entail vaccination such as deworming medicine, and iron and folic acid supplementation.

#### Actions taken: intensify community engagement

In response to declining vaccination coverage and disease outbreaks, respondents explained how the DOH implemented house-to-house campaigning to track defaulters or those who did not complete scheduled vaccinations. HCWs were asked to visit communities and administer vaccines directly at families’ doorsteps. Respondents also reported conducting several engagement meetings with local government stakeholders (i.e. provincial governors, mayors, and local leaders) to ensure their buy-in for catch-up vaccination campaigns. In these roundtable discussions, policymakers attempted to create a ‘*sense of local ownership of the program*’ [CO, 5 years] by asking for and listening to recommendations of local stakeholders.

### The fallout deepens amid the COVID-19 pandemic

#### Challenges to maintain vaccination structures and COVID-19 vaccination discourses

Respondents spoke at length about the impact of the COVID-19 pandemic on a vaccination program that was still reeling in the aftermath of the Dengvaxia scare. In the eyes of respondents, the country had not yet overcome measles and polio outbreaks of previous years, and the house-to-house polio campaign had not been completed, which made the timing of COVID-19 particularly unfortunate.

Community lockdowns resulted in a ‘*logistical nightmare*’ [RN, 2 years], placing heavy burdens on already-strained human resources. For house-to-house vaccination campaigns, which heavily rely on local support and coordination, respondents explained how entering communities became increasingly problematic, with some local leaders banning everyone including HCWs from entering out of concerns regarding SARS-CoV-2 transmission. Respondents explained that HCWs were facing parents who remained afraid of the HCWs potentially transmitting COVID-19 when administering oral polio vaccine, or that the vaccine itself could transmit the SARS-CoV-2 virus.

Additionally, respondents noticed that hope for and development of a COVID-19 vaccine reshaped broader vaccination narratives among Filipinos. Respondents recounted experiencing widespread public excitement on TV or social media regarding a potential vaccine (at the time of data collection, no vaccine had been approved for national rollout). They felt that this indicated how the experience of a viral pandemic led to a greater awareness in the general public regarding why vaccines are important and how they benefit everyone. However, respondents were worried that a COVID-19 vaccine might meet the same fate as the dengue vaccine, urging caution and careful communication and rollout.

### Actions taken: develop facility workarounds

Respondents shared how policies were reshaped to continue facility-based delivery of vaccination services while adhering to basic infection control measures. Measures taken included changing the facility set-up (i.e. social distancing, outside waiting areas), implementing a skeletal staff work schedule (i.e. work-from-home, four-day compressed work week, staggered working hours), and imposing a strict infection, prevention, and control protocol (i.e. wearing of masks and face shields, use of alcohols and disinfectants). In cases where community entrance for vaccination campaigns remained feasible, protective procedures were incorporated into standard vaccination procedures (i.e. SARS-CoV-2 testing of HCWs before proceeding into communities, proper donning of personal protective equipment).

We present key quotes for each of the themes of policymakers’ experiences highlighted above in Table 2.

**Table 2.** Experiences and perceptions of policymakers on vaccine scare in the Philippines.

Themes	Illuminating Quotes
**The scare – the case of Dengvaxia**	
Loss of trust, distorted narratives and personal guilt	‘So, that time [during the Dengvaxia controversy] we had to move around every school. I was with people from the Department of Health (DOH), whom I didn’t know, and only met there and then … It was hard … being the punching bag of somebody and I don’t like that. No one should lie and just tell the parents the truth, that it was not the Department of Education (DepEd)’s program alone. It was the program of the government … it was a tripartite [of DepEd, DOH and Executive Branch of the government], a collaboration but the main decisionmakers are not from DepEd right?’ [MD, 20 years]
	‘I felt ridiculous for one thing to see how the government allowed non-scientific people to distort the narrative … That has damaged the reputation of the Department of Health, the programs it has managed … including those who were involved in the decision making …. We felt very ridiculous and betrayed by those who proclaim to be experts in public health … and they have caused great disservice by alluding to something that is not true.’ [MD, 4 years]
	‘The rumors spread very quickly … the social media facilitated the spread of inaccurate information that isn’t helpful. It has been very damaging … and we suffered a lot.’ [MD, 2 years] ‘Oh my God. Oh my! I got pissed with those [false information]. The TV is exaggerated. … You can see the reaction of the mothers “Hey! Let’s not get vaccinated, that is deadly”. You know, these rumors that are not true spread quickly.’ [MD, 16 years]
**The fallout unfurls– domino and spillover effects**	
Spillover effects to other vaccine programs and beyond vaccination	‘So, ah dengue vaccine had a negative impact on the overall national immunization program in the Philippines. But I don’t want to take dengue vaccine as a sole issue. … However, I agree to some extent it has caused some public distrust.’ [MD, 20 years]
	‘But of course, there is really fear, we even see its effect in our deworming program … Our coverage was 63%, then there was Dengvaxia, it goes down to 32%. … imagine how our deworming program became the collateral damage.’ [MD, 16 years]
**The fallout deepens amid the COVID-19 pandemic**	
Challenges to maintain vaccination structures and COVID-19 vaccination discourses	‘In some areas, vaccination coverage declined by more than 50% [during COVID-19]. … Because of the community lockdown, the health center cannot deliver wider services … the parents cannot go out of their home; health workers also cannot move freely.’ [MD, 29 years]
	‘So, with the COVID vaccine introduction, we must be very careful … COVID vaccine also can suffer the same fate as the dengue vaccine. … I am a little worried that people again will distrust again the routine immunization, also because antivaccine group might use this opportunity.’ [MD, 20 years] ‘If there is any consolation in this COVID pandemic, it is that people are more aware of the vaccine. … Hopefully it will bring back that kind of confidence to all vaccines.’ [MD, 2 years]

### Lessons learned and taking actions – policymakers’ perspectives on rebuilding trust and vaccine confidence

With regard to lessons learned and plans and recommendations for future action, respondents noted a need to better strategize and align vaccine innovations to the overall goal of a healthy population. We present overarching themes in relation to actions to be taken which are accompanied by salient quotes (see Table 3).

**Table 3.** Strategies to bolster vaccine uptake in the Philippines.

Key Findings	Illuminating Quotes
**Rebuilding trust in the government**	
Transparency	‘[The government] should have only one voice, and be open … You have to be sincere, provide a level of openness and sympathy to the people, that is very important at the service delivery. We should not force the people … we should not hide some information to the people you know, we should be open.’ [MD, 20 years]
**Clear vaccine messaging**	
Tailor-fit communication (highlight benefits, discuss the risks)	‘I think what you should talk about is how are we going to communicate risks to the public. So, you don’t need to tell the mothers each issue and all detail about the molecular issues, you know … You make them scared. But you cannot hide those, so you must find the balance … Again, it all depends on the mother’s knowledge.’ [MD, 20 years]
Traditional media education	‘We should educate the media people first. If the media become aware and educated, then they will communicate correct messages. Because they know what the truth is … The dangerous thing is when these people don’t know what the truth is. They are just like an intermediary between the government and the people. That is the risk.’ [MD, 16 years]
Use of social media	‘One is to innovate the marketing skills, making it simpler, easy to understand. And they [the World health organization] really has to convey a good picture how beneficial it [the COVID-19 vaccine] will be. That can be turned into a presentation ... maybe delivered via social media or Facebook.’ [MD, 5 years]
Engage with religious leaders, celebrities and/or public figures	‘You [should] consider the local context and make use of specifically local influencers. So, it could be the mayor or maybe the wife of the mayor or the governor. It will help a lot.’ [MD, 16 years] ‘We sit down with the religious leaders [and] discuss the importance of vaccines … in ensuring the communities’ safety. Then, we arrive at an agreement. Once we have that agreement, mothers agree for vaccination.’ [RN, 2 years]
UnderstandVH groups	‘The first thing you need to do is read about the antivaccine group … Read about them very well, what they are saying. What they do is, they twist science … so that is the place where you must go and check correctly. You must answer that point. Don’t go and say this is a scientific vaccine, that this is based on science.’ [MD, 20 years]
**Fostering HCWs’ communication skills**	
Risk communication	‘The people have great respect for the clinicians. And remember when there are problems with the vaccines, they go first to the hospital … When the doctor says “Oh, this is because of the vaccine”, game over … we are done. The doctor is knowledgeable to properly advice, but you know the doctor should nicely communicate it … For me, the number one priority is our clinicians.’ [MD, 20 years]

#### Rebuilding trust in the government

Respondents demanded a comprehensive strategy to rebuild the trust of the people in the government. Some highlighted that the government should have one clear and consistent voice, as conflicting messages might cause doubts and apprehension towards all governmental programs, including but not limited to the vaccine programs. Respondents also explained how regaining trust is difficult and would take time. Transparent communication of all processes, from vaccine components and trial results (specifically for new vaccines) to national procurement efforts, would facilitate widespread vaccination uptake.

#### Clear vaccine messaging

Respondents explained that changing beliefs and behaviors of vaccine hesitant parents is challenging, and that showing them the consequences of not vaccinating (i.e. by graphic stories or pictures) might be a useful approach. However, other respondents cautioned not to make the messaging too fearful as it might cause more harm than good.

Regarding characteristics of effective communication, respondents highlighted the importance of creative yet clear messages, and the need for educational material that could be acceptable to end-users in various contexts (e.g. for facility-based information, as part of house-to-house campaigns, or for independent reading at home). Respondents also highlighted the potential of social media, especially Facebook, as channels which are used by a majority of the population, potentially combined with the engagement of influential people to maximize visibility and reach. Among others, respondents recommended exploring animated videos, real-life documentaries, pamphlets, and presentations in the form of infographics to reach hesitant parents.

Respondents also conveyed the importance of engaging and conversing with religious leaders, as their role as trusted agents could lead to increased confidence among previously hesitant parents and increased motivation among people involved in vaccination rollout. Considering the role of misinformation in vaccine scares, respondents called for traditional media and journalists to be actively involved in conscious vaccination communication and in confidence recuperation efforts.

#### Fostering HCWs’ communication skills

Respondents discussed the need for continued training to expand HCWs’ communication approaches, including special risk and science communication training for all HCWs delivering health services (especially relating to vaccines). This was particularly relevant in the context of respondents’ concern that if medical doctors (whom the community really trusts) were to be perceived as doubting vaccination effectiveness and safety, this could further exacerbate community VH.

## Discussion

Our study highlighted perceptions of policymakers who identified several challenges to current implementation efforts of the childhood vaccination program, especially due to vaccine controversies associated with the Dengvaxia scare and the current COVID-19 pandemic. Our results suggest that the presence of large-scale vaccine scares is often exacerbated by various contextual and political forces exerting pressure not only on vaccine confidence, but also on general health programming. Although policymakers have developed strategies and policies to cope with past vaccine scares and current vaccination challenges to bolster vaccine uptake, further efforts are needed in the context of evolving threats.

Our findings on spillover effects stemming from mistrust in one particular intervention (in our case, introduction of a new vaccine) or institution, and the impact they can have on other health-related factors, resonates with reports from other contexts [38,39]. In particular, a review found that after a health system-wide shock, as evident in the Ebola outbreak in Sierra Leone, distrust in the government lingered, resulting in decreased vaccination rates spilling over onto the general health system (i.e. under-utilization of health services), and vice versa [38]. In the case of the Philippines, however, the patterns of mistrust and spillover effects reflect the particular characteristics of the vaccine scare: with Dengvaxia being introduced by the DOH, but rolled out as a part of the school-based interventions under the patronage of the DepEd, respondents reported spillover effects of mistrust regarding school-based health interventions [22]. As a result, the DepEd experienced a more profound discrediting as compared to the DOH, leading to considerable tensions between these institutions. Understanding the extent to which spillover effects alter the trust in other health programs and across governmental institutions will be essential to ensure viable solutions and to prevent this from re-occurring.

Respondents in our study saw social media as playing a decisive role in spreading misinformation or emotionalized images (e.g. children allegedly dying from the vaccine) in the Philippines, leading to widespread suspicion of vaccines and the government [22,23]. Existing evidence suggests a relationship between social media and proliferation of public doubts, and the decline of vaccine coverage [40–42]. In spite of concerted efforts to redirect the public towards reliable sources for verified information, social media has transformed the way people communicate globally. These dynamics have led the WHO to call for a global movement to promote accessibility to health information and to create solutions to counter the spread of misinformation in traditional and social media platforms [43]. The concept of communication efforts being designed for, and targeted at, particular groups and cultural contexts reflects the current tenor in the literature [44,45], but evidence on successful implementation of large-scale communication campaigns for VH is so far limited [46]. Considering evidence that HCWs’ recommendations and communication are among the most efficient ways to increase vaccination uptake [46], our respondents' recommendations to improve HCWs and physicians communication skills and building emergency risk communication is paramount. Such efforts could for example include trainings for providers on how to engage VH parents in an open empathic conversation in the hope of assisting them to develop their own vaccination motivations [47] and building emergency risk communication into the public health system in general is paramount. Furthermore, our findings call for more insights into how we can work with HCWs – appreciating that they too might have reservations about vaccines, that they struggle when encountering VH parents, and that they are seeking to receive and share evidence on what works.

Our finding that vaccine scares such as Dengvaxia were weaponized for political purposes is particularly relevant in the context of growing evidence suggesting a connection of political views and the rise of VH [48,49]. This for example has also been highlighted in Nigeria, where political instability has created a lack of trust in governmental institutions generally, which has undermined the state vaccination program’s credibility [49]. A survey in 28 European countries similarly has emphasized that histories of no vaccination for the past five years because of safety concerns are linked to low trust in the local and national governments [50]. Evidence suggests that political and moral outlooks may significantly disrupt vaccine uptake, and that the role of policymakers is critical in all aspects of the VH continuum [48,50]. The presence of national health policies that are transparent and clear are particularly suited for reaching those most susceptible to VH, and building in feedback at every stage to bridge communication gaps is of great importance in all aspects of reviving vaccine uptake.

The fallout of the Dengvaxia scare in the Philippines is particularly noteworthy in contrast to the situation in the four other countries (i.e. Brazil, Mexico, El Salvador and Costa Rica) where Dengvaxia was introduced [51], all of which have not experienced the same level of public outcry. Following the emergence of evidence regarding potential side effects, some of these countries temporarily halted Dengvaxia rollout but resumed vaccination programs once evidence was available that a risk only applied to individuals without prior dengue infection and protocols had been refined accordingly [51]. Similarly, the WHO listed Dengvaxia as an ‘essential medicine’ in 2019 [52] and 20 countries, including the US and countries in the European Union, licensed and continue to use Dengvaxia for individuals with prior exposure to the dengue virus [53]. Few studies have examined why the developments in the Philippines differed so drastically from those in many other countries, arguing that political interests following a change in government and allegations of corruptions targeted at the previous government contributed to the widespread vaccine scare [23,29,31]. Our study adds to this discourse by underscoring the role of social and traditional media in shaping the public’s vaccine-related decisions and igniting polarized understandings about vaccines. At the same time, while the alleged fallout of the vaccine scare with regard to the public’s trust in vaccines was dramatic, political consequences proved limited in at least one sense: none of the original charges against government or pharmaceutical company officials have resulted in convictions; this situation of fomenting fear but leaving no party accountable may have sparked additional frustration among the Filipino public [54]. We encourage further case study research examining how contextual differences may have underpinned markedly different experiences across countries.

This study has limitations. First, we highlight that due to COVID-19 restrictions, our interviews were conducted via online platforms, which might have affected the nature of information shared and level of rapport built [34]. Additionally, several overlapping vaccination discourses in the Philippines (COVID-19, Dengvaxia) emerged, which challenges the identification of clear relationships between specific discourses and facets of VH. We also emphasize that although all respondents were working in the field of vaccination, not all policymakers had practical experience with VH intervention development; some recommendations therefore may not be experience-based. Finally, we highlight that there may have been an element of social desirability bias in respondents’ answers, as a majority of the research team are based at RITM and the subject studied is one of professional sensitivity. We hope that we have mitigated most bias by acknowledging the process of data co-construction between the respondents, the interviewers and those that analyzed the data by building our codes and themes with this in mind. We did our best to ensure that respondents understood their answers were confidential, that there were no right or wrong answers and that we were interested in their personal opinion.

## Conclusion

Our study explores how the Dengvaxia vaccine scare and current COVID-19 vaccination-related challenges have shaped narratives about vaccines, public confidence in vaccines, health programming, and the health system in the Philippines. According to policymakers, the vaccine scare has continuously affected parental decision-making and trust and resulted in negative spillover effects within other health programs. Actionable recommendations from our data on how to rebuild vaccine confidence include established guidance (transparency, use of traditional and social media for education purposes) and culturally sensitive and novel approaches (targeted offers for journalists, the involvement of local influencers), highlighting the importance of considering the views and experiences of local policymakers in the design and research of targeted health interventions. Rebuilding trust and buy-in for vaccines globally is more necessary - albeit more challenging - than ever.

## Paper context

Policymakers are now more than ever in the challenging position to negotiate the fallout of vaccine scares – such as the proliferation of misinformation – while addressing existing challenges in vaccination rollout. This paper can guide academics, implementers, and policymakers who are considering fostering vaccine confidence, improving future vaccination rollout and leveraging ways on rebuilding vaccine program resilience and community buy-in to vaccination in the Philippines and similar contexts.
